# The Identification of Peptide Inhibitors of the Coronavirus 3CL Protease from a *Fucus ceranoides* L. Hydroalcoholic Extract Using a Ligand-Fishing Strategy

**DOI:** 10.3390/md22060244

**Published:** 2024-05-27

**Authors:** Luiz Antonio Miranda de Souza Duarte Filho, Cintia Emi Yanaguibashi Leal, Pierre-Edouard Bodet, Edilson Beserra de Alencar Filho, Jackson Roberto Guedes da Silva Almeida, Manon Porta Zapata, Oussama Achour, Hugo Groult, Carlos Arthur Gouveia Veloso, Claudio Viegas Júnior, Nathalie Bourgougnon, Laurent Picot

**Affiliations:** 1 Littoral Environnement et Societés (LIENSs), UMRi CNRS 7266, La Rochelle Université, 17042 La Rochelle, France; lmiranda@etudiant.univ-lr.fr (L.A.M.d.S.D.F.); manon.porta@etudiant.univ-lr.fr (M.P.Z.); oussama.achour@univ-lr.fr (O.A.); hugo.groult@univ-lr.fr (H.G.);; 2 Unité de Génie Enzymatique et Cellulaire, UMR CNRS 7025, Université de Picardie Jules Verne, 80039 Amiens, France; cintia.leal@u-picardie.fr; 3 Plateforme D’analyse Haute Résolution des Biomolécules, UMR CNRS 7266 LIENSs, La Rochelle Université, 17042 La Rochelle, France; pierreedouard.bodet@univ-lr.fr; 4 Department of Pharmacy, Universidade Federal do Vale do São Francisco, Petrolina 56304-205, PE, Brazil; edilson.beserra@univasf.edu.br; 5 Núcleo de Estudos e Pesquisas de Plantas Medicinais (NEPLAME), Universidade Federal do Vale do São Francisco, Petrolina 56304-205, PE, Brazil; jackson.guedes@univasf.edu.br; 6 Institute of Chemistry, Federal University of Alfenas, Alfenas 37130-000, MG, Brazil; claudio.viegas@unifal-mg.edu.br; 7 Laboratoire de Biotechnologie et Chimie Marines, LBCM, Université Bretagne Sud, EMR CNRS 6076, IUEM, 56000 Vannes, France; nathalie.bourgougnon@univ-ubs.fr

**Keywords:** antiviral activity, coronavirus, fucus, ligand fishing, macroalgae, peptide, protease, SARS-CoV-2, seaweed

## Abstract

Brown seaweeds of the *Fucus* genus represent a rich source of natural antiviral products. In this study, a *Fucus ceranoides* hydroalcoholic extract (FCHE) was found to inhibit 74.2 ± 1.3% of the proteolytic activity of the free SARS-CoV-2 3CL protease (3CLpro), an enzyme that plays a pivotal role in polyprotein processing during coronavirus replication and has been identified as a relevant drug discovery target for SARS- and MERS-CoVs infections. To purify and identify 3CLpro ligands with potential inhibitory activity using a one-step approach, we immobilized the enzyme onto magnetic microbeads (3CLpro-MPs), checked that the enzymatic activity was maintained after grafting, and used this bait for a ligand-fishing strategy followed by a high-resolution mass spectrometry analysis of the fished-out molecules. Proof of concept for the ligand-fishing capacity of the 3CLpro-MPs was demonstrated by doping the FCHE extract with the substrate peptide TSAVLQ-pNA, resulting in the preferential capture of this high-affinity peptide within the macroalgal complex matrix. Ligand fishing in the FCHE alone led to the purification and identification via high-resolution mass spectrometry (HRMS) of seven hepta-, octa-, and decapeptides in an eluate mix that significantly inhibited the free 3CLpro more than the starting FCHE (82.7 ± 2.2% inhibition). Molecular docking simulations of the interaction between each of the seven peptides and the 3CLpro demonstrated a high affinity for the enzyme’s proteolytic active site surpassing that of the most affine peptide ligand identified so far (a co-crystallographic peptide). Testing of the corresponding synthetic peptides demonstrated that four out of seven significantly inhibited the free 3CLpro (from 46.9 ± 6.4 to 76.8 ± 3.6% inhibition at 10 µM). This study is the first report identifying peptides from *Fucus ceranoides* with high inhibitory activity against the SARS-CoV-2 3CLprotease which bind with high affinity to the protease’s active site. It also confirms the effectiveness of the ligand-fishing strategy for the single-step purification of enzyme inhibitors from complex seaweed matrices.

## 1. Introduction

The COVID-19 pandemic and related coronavirus infections remain a challenging global health issue. With the emergence of new variants, insufficient immune responses in vaccinated individuals, global vaccine distribution disparities, and persisting infection rates, the development of effective antiviral treatments is still a priority for researchers and healthcare professionals around the globe [1,2]. In this context, natural marine products represent an extremely valuable source of antiviral drug candidates due to their diversity and unique chemical scaffolds [3].

One of the main enzymes required for SARS-CoV-2 replication is the 3-chymotrypsin-like cysteine protease (3CLpro). This protein plays a key role in polyprotein processing, cleaving polyprotein 1ab at 11 sites and releasing the mature viral proteins necessary for viral assembly and replication. The 3CL protease catalyzes the cleavage of its substrates at the peptide bond adjacent to a glutamine residue on the C-terminal side (Q↓) [4]. Additionally, it possesses the capability to hydrolyze ester bonds between glutamine and subsequent molecules in the C-terminal position, as demonstrated by the chromogenic substrate TSAVLQ-pNA. The structural conservation and essential function of the 3CLpro among coronaviruses make it an attractive target for the development of antiviral drugs [5]. In this context, the study of marine plants and macro- and microorganisms with an intention to identify new molecules inhibiting this enzyme is a relevant strategy for the discovery of drugs likely to have a significant impact on the prevention and treatment of COVID-19 [5].

The identification of new natural products with specific biological activities requires fast and efficient screening and purification methods. Ligand fishing, a powerful technique used for the affinity-based isolation of target protein ligands, offers a valuable approach in the field of drug discovery. Functionalized magnetic beads, for instance, provide a versatile tool for capturing potential lead compounds from complex natural product matrices. By combining the unique characteristics of natural products and the versatility of ligand-fishing techniques, the discovery of new therapeutic agents can be optimized [6].

Some species from the *Fucus* genus are known for their diverse biological activities, which include antiviral effects [7]. However, *F. ceranoides* is poorly studied in this sense. In this study, we aimed to investigate the antiviral potential of a *Fucus ceranoides* L. hydroalcoholic extract against the COVID-19 main protease by employing a ligand-fishing strategy with 3CLpro-grafted magnetic particles (3CLpro-MPs). Through a multidisciplinary approach including enzymology and analytical chemistry, we identified new lead marine peptides with high binding affinities for the 3CLpro active site and protease inhibitory activity that may contribute to the development of effective preventive or therapeutic formulations against COVID-19.

## 2. Results

### 2.1. Inhibitory Effects of FCHE and PF-00835231 against Free 3CLpro

**Figure 1:**
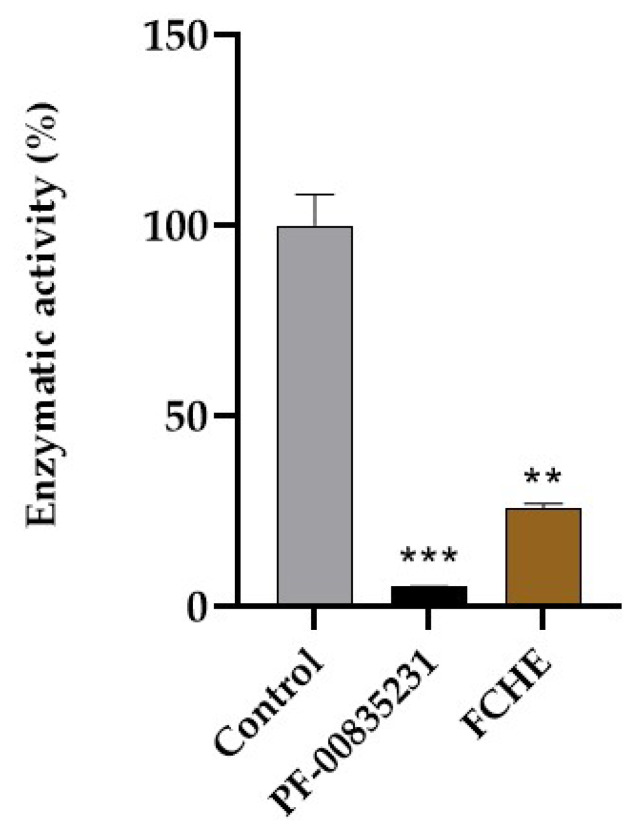
Normalized free 3CLpro enzymatic activity under control conditions (100% enzymatic activity) or in the presence of PF-00835231 at 1 µM or FCHE at 100 μg·mL^−1^. ** Represents a significant difference (*p* < 0.01) according to unpaired Student’s *t*-test (FCHE vs. control). *** Represents a significant difference (*p* < 0.001) according to unpaired Student’s *t*-test (PF-00835231 vs. control).

FCHE 100 μg·mL^−1^ inhibited 74.2 ± 1.3% of the proteolytic activity of the SARS-CoV-2 3CLpro. Additionally, the synthetic 3CLpro inhibitor PF-00835231 (1 μM) was able to inhibit 94.9% of the 3CLpro control’s enzymatic activity (Figure 1). Given that the molecular weight of PF-00835231 is 472.53 g·mol^−1^, a 1 µM solution equates to 472.53 ng·mL^−1^. This concentration, resulting in 94.9% inhibition, is to be compared with the FCHE concentration of 100 µg·mL^−1^, which was 212 times less concentrated and induced a 20.7% lower inhibition. This highlights the need to purify and concentrate the bioactive compounds in the extract by a minimum factor of 250 to achieve a comparable inhibitory activity.

### 2.2. Enzymatic Activity of 3CLpro-Grafted Magnetic Particles (3CLpro-MPs)

After proceeding with immobilization, the enzymatic activity of the 3CLpro-MPs was verified as described in the Materials and Methods section. Normalized data showed that the 3CLpro-MPs’ enzymatic activity (139.1 ± 5.9%) was higher than that of the free 3CLpro (100 ± 6.5%). This observed enhancement in activity could potentially be attributed to an elevated substrate concentration within the microenvironment surrounding the enzyme-grafted magnetic particles, but the spectrophotometric enzymatic assay did not allow us to test this hypothesis.

**Figure 2:**
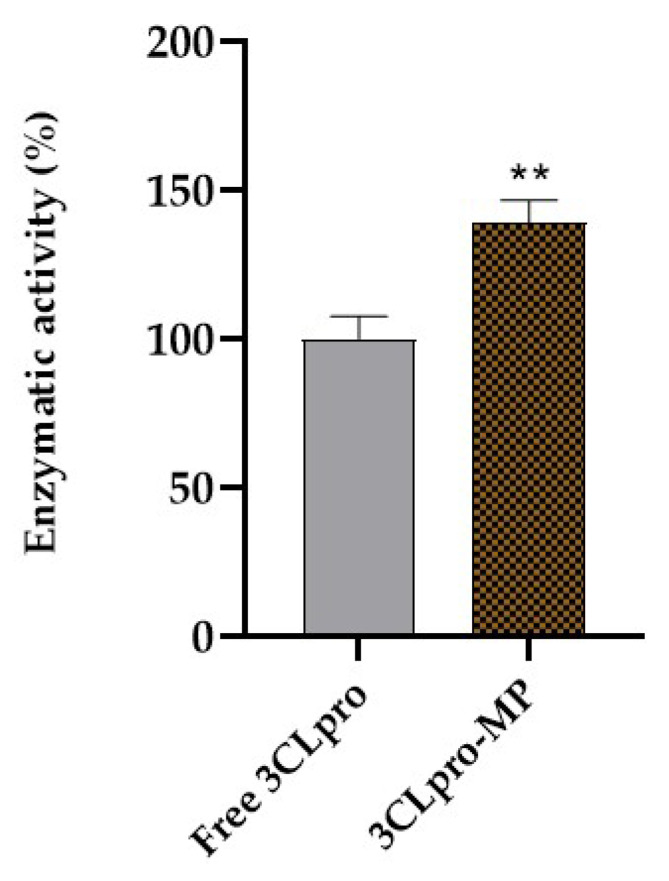
Normalized 3CLpro-MP enzymatic activity in relation to enzymatic activity of free 3CLpro (100%). ** Represents a significant difference (*p* < 0.01) according to unpaired Student’s *t*-test (free 3CLpr vs. 3CLpro-MP).

The MPs alone presented no enzymatic activity, so they are not represented in the following graph (Figure 2).

### 2.3. Dynamic Light Scattering (DLS) and Zeta Potential of 3CLpro-MPs

According to data obtained from a DLS analysis, there was an increment of about 224 nm in the mean hydrodynamic volume of the magnetic particles (MPs) after the immobilization of 3CLpro compared to the MPs alone (Figure S10). The MPs presented a polydispersity index (PDI) of 0.1622 ± 0.026, whereas the 3CLpro-MPs presented a PDI of 0.1298 ± 0.058. The zeta potential measures revealed a surface charge of −35.06 ± 0.493 mV for the MPs and −35.06 ± 0.321 mV for the 3CLpro-MPs (Table 1).

### 2.4. Immobilization Efficiency

The immobilization efficiency of the 3CLpro on the surfaces of the magnetic particles was calculated to be 63.5% and 67.7% after 1 and 2 h of incubation, respectively.

### 2.5. Proof of Concept of LIGAND Fishing with the Chromogenic Substrate (TSAVLQ-pNA)

**Figure 3:**
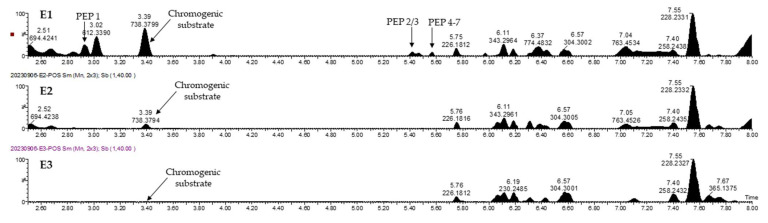
LC-HRMS chromatograms (expressed as a percentage of the highest peak’s intensity) of eluants after elution steps E1, E2 and E3. Mass spectrometry chromatograms obtained in positive polarity from the three eluates E1, E2 and E3.

After doping the FCHE with 10 μM of TSAVLQ-pNA, we observed that the chromogenic substrate was successfully fished by the 3CLpro-MPs(a recovery of 46.8% of the initial amount in E1, 12.2% in E2, and 4.7% in E3). Moreover, the substrate was found only in the elution steps (E1, E2, and E3), with decreasing concentrations from one elution step to the next. Figure 3 shows the LC-HRMS chromatograms of the three elution steps.

### 2.6. Inhibitory Effect of E1 + E2 + E3 Mix of Eluents against Free 3CLpro and HRMS Identification of Peptides in Eluate Mix

**Figure 4:**
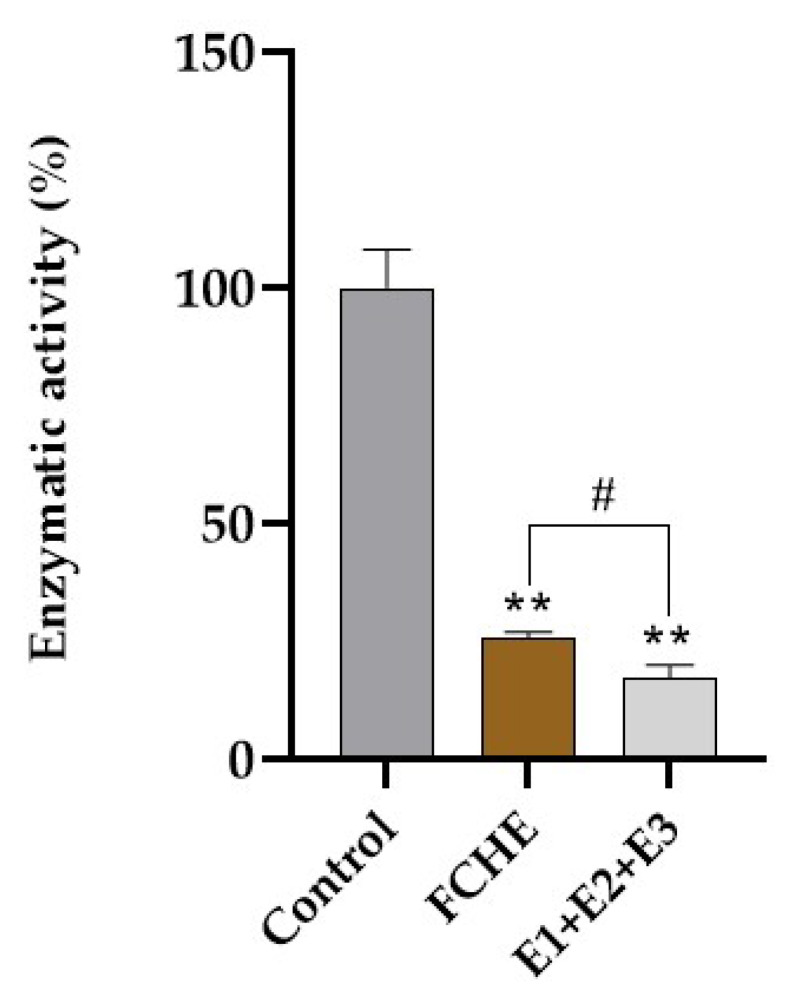
Normalized free 3CLpro enzymatic activity under control conditions (100% enzymatic activity) or in the presence of the FCHE at 100 µg·mL^−1^ or the ligand-fishing eluate mix E1 + E2 + E3. ** represents a significant difference (*p* < 0.01) in relation to the control, according to the unpaired Student’s *t*-test. # Represents a significant difference (*p* < 0.01) according to the non-paired Student’s *t*-test (FCHE vs. E1 + E2 + E3).

The eluate mix (E1 + E2 + E3) recovered after ligand fishing in the FCHE (without doping with the chromogenic substrate) inhibited 82.7 ± 2.2% of the free 3CLpro’s activity, a value significantly higher than the inhibitory effect of the complete FCHE (Figure 4).

An HRMS analysis of the eluate mixture identified the presence of over 30 peptides. Some of these contained isoleucine or leucine that were not distinguished by the HRMS analysis, and only one over two may be actually present in the extract (PEP2 and PEP3 and PEP 4, PEP 5, PEP6, and PEP7). The peptides exhibiting the highest ion abundances in the MS analysis lacked glutamine in their sequences, indicating they were unlikely substrates for the enzyme. Within the peptide mixture were dipeptides like VE, TV, and SI/L alongside longer peptides and amino acids, all likely contributing to the overall inhibitory activity of the eluate. To isolate a subset of peptides with clear sequences from this mixture, which potentially possess a high affinity for the 3CL protease active site, we targeted seven hepta/octa- or decapeptides (Table 2) whose affinity for the active site was assessed by a molecular docking analysis. The results of an HRMS analysis of these 7 peptides are presented in the Supplementary Materials (Figures S1–S3). This selection was informed by previous data indicating the inhibitory effect of peptides or peptidomimetic agents with five or more amino acids against both SARS-CoV-1 and SARS-CoV-2 3CL proteases. For example, thymopentin, a clinically approved drug consisting of the pentapeptide RKDVY, demonstrates high inhibitory potential against the 3CL protease from SAR-CoV-2 [8]. Notably, the N-terminal extremity of this peptide bears a resemblance to the peptides that were found and chosen in our study for docking studies, with two hydrophobic amino acids, and the final residue Y (PEPs 1-3).

### 2.7. Molecular Docking

**Figure 5:**
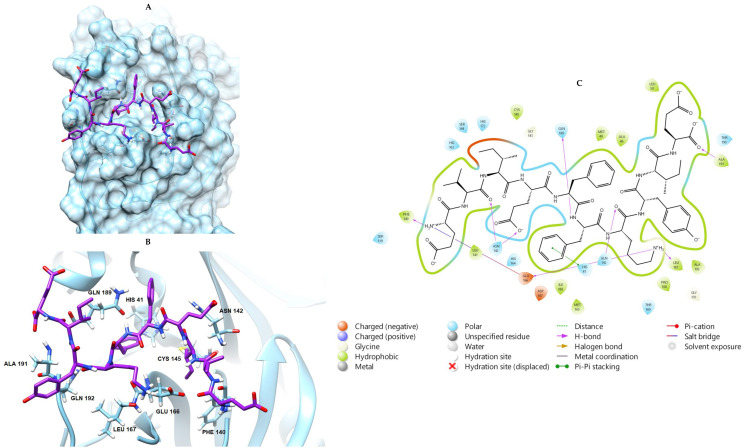
The most stable conformation of PEP 4, the peptide showing the highest docking score at (**A**) the Van der Waals surface, (**B**) the binding site of the co-crystallized peptide aldehyde ligand with the highest docking score (pdb 3AW0), and (**C**) a 2D representation of possible interactions.

In an initial step, a redocking procedure was performed with the co-crystallographic peptide ligand at the active site of the 3CLpro. In order to achieve the best RMSD, various parameters were tested in the GOLD program, and the best result was obtained using the default with the exception of the ligand flexibility, which was set to 200%. In this configuration, it was possible to obtain an RMSD of 1.889 Å. Molecular docking simulations of the seven peptide sequences fished from the FCHE revealed a better affinity for the 3CLpro than the co-crystallographic ligand, as shown in Table 2. All peptides identified showed a good binding capacity in the 3CLpro’s active site and interactions with neighboring amino acids. Plausible non-covalent interactions within the active site of the protease involved an electrostatic attraction for His 41 and a hydrophobic interaction with Cys 145. Modeling also revealed the possibility of several other polar (hydrogen bonds and electrostatic) and hydrophobic interactions with amino acid residues located outside of the active site (see Figure 5 for PEP4, the peptide showing the best affinity score, and Figures S4–S9 for the other peptides).

### 2.8. An Evaluation of the Inhibitory Activity of Synthesized Peptides (PEPs 1-7) Identified through the Ligand-Fishing Approach

**Figure 6:**
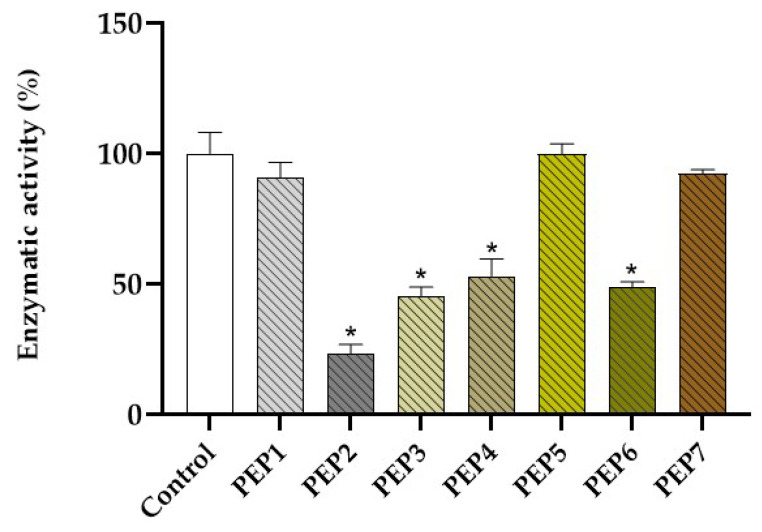
Normalized free 3CLpro enzymatic activity under control conditions (1% DMSO) or in the presence of PEPs 1-7 (10 µM and 1% DMSO). * Represents a significant difference (*p* < 0.01) in relation to the control according to a one-way ANOVA followed by Dunnett’s multiple-comparison test.

After conducting docking simulations, we individually assessed the synthesized peptides (1-7) under the same experimental conditions utilized in prior assays. Our findings indicated that PEP 2 (76.8 ± 3.6%), PEP 3 (54.8 ± 3.5%), PEP 4 (46.9 ± 6.4%), and PEP 6 (51.2 ± 1.9%) significantly inhibited the free 3CL protease in vitro (using a one-way ANOVA followed by Dunnett’s multiple-comparison test, *p* < 0.01). Conversely, PEP 1 (9.3 ± 5.7%), PEP 5 (0.01 ± 3.8%), and PEP 7 (7.6 ± 1.3%) induced non-significant inhibition against the free 3CL protease (Figure 6).

## 3. Discussion

The COVID-19 main protease, which is essential for viral replication, consists of two subunits that cleave eleven sites on the non-structural polyproteins 1a and 1ab, releasing functional proteins necessary for viral assembly [9]. Consequently, inhibiting this enzyme is a relevant strategy to block viral multiplication.

Marine organisms possess significant potential for innovation in the field of drug discovery [10]. Brown algae from the *Fucus* genus, for instance, have been extensively explored for their diverse biological activities [7]. However, the species *Fucus ceranoides* L. has not provided many bioactive compounds so far. In this work, we demonstrated for the first time that a hydroalcoholic extract of *F. ceranoides* (FCHE) was able to significantly reduce the protease activity of the 3CLpro in vitro (Figure 1). It is important to note that this effect is very unlikely to be provoked by fucoidans since they precipitate in ethanol [11]. This class of biological active saccharides present in the *Fucus* genus are known for their versatile biological activities, including an anti-COVID-19 activity [12]. However, other metabolites may have acted as 3CLpro inhibitors, such as peptides [13,14].

In order to check if the 3CLpro immobilization procedure was successful and did not impair enzymatic activity, we proceeded with an assessment of the protease activity of the 3CLpro-MPs. According to our data, the 3CLpro increased its enzymatic activity after immobilization by 39.1% in relation to the free 3CLpro (Figure 2). This phenomenon can be explained by different mechanisms: immobilization on solid supports can change an enzymatic conformation to a more rigid form due to multipoint attachment (covalent bonds), which may result in more accessible active sites. However, we did not determine the orientation of the 3CLpro after immobilization, though we assumed it favored its proteolytic activity. Moreover, immobilization on magnetic particles may reduce the particle aggregation ability, which also contributes to the accessibility of the substrate to the catalytic site [15,16]. In a study conducted by Yin and colleagues, α-chymotrypsin was immobilized on calcium phosphate nanoflowers, and they observed that the proteolytic activity was 266% of the activity of the free enzyme [17]. In another study, the proteolytic activity of α-chymotrypsin immobilized on the surface of a cellulose magnetic particle was also augmented [18].

Dynamic light scattering (DLS) is a technique employed in the characterization of particles in suspensions [19]. In the context of magnetic-particle-based ligand fishing, DLS can provide important information about particle size distribution that is critical to the efficient capture of bioactive molecules [20]. According to our data, there was an increase of about 224 nm in the mean particle size of the 3CLpro-MPs in relation to the magnetic particles alone, indicating the presence of the 3CLpro on the surfaces of the particles (Figure S10). Intuitively, since the molecular weight and diameter of proteins may vary drastically, the increment in particle size after immobilization may also do so. However, there is no apparent proportionality between protein molecular weight and an increase in the mean size of particles in suspension. Indeed, an increment of 10–20 nm in the mean size of a particle can occur as a result of the immobilization of a protein of a few hundred to tens of thousands of Daltons [21,22]. Moreover, PDI measurements revealed that both the MPs and 3CLpro-MPs presented a monodispersed behavior with values below 0.2, which is important for efficient ligand fishing [20,23].

After validating the 3CLpro-MPs as functional bait, we proceeded with the ligand-fishing experiment. Using this strategy, we were able to identify and characterize through HRMS some peptide sequences present in the eluates (E1, E2, and E3) (Table 2 and the Supplementary Materials). In the enzymatic tests, the mix of eluates (E1 + E2 + E3) was able to inhibit 82.7% of the enzymatic activity of the free 3CLpro compared to the control condition against 74.2% inhibition by the whole FCHE. This suggests that the 3CLpro-MPs were able to fish the active molecules in the FCHE that were mainly responsible for 3CLpro’s inhibitory activity. Indeed, peptides have been vastly studied due to their potential to inhibit coronavirus replication [24,25], and it makes sense to identify peptides as protease inhibitors. In a study conducted by Wang and colleagues, a virtual screening of clinically approved drugs with the SARS-CoV-2 3CLpro as a target was performed. They found that thymopentin, a pentapeptide consisting of RKDVY, presented the best score among 21 approved drugs [8]. Peptides may serve as important chemical scaffolds for the discovery of new anti-COVID-19 agents, notably aldehyde derivatives [26].

From this perspective, we carried out the molecular docking of the seven peptides fished from the FCHE (PEPs 1-7) at the active proteolytic site of the 3CLpro. Interestingly, the docking scores for all selected peptides were higher than that of the co-crystallographic ligand, suggesting that they have a superior binding affinity for the target compared to the validated inhibitor (Table 2). Although the binding affinity does not allow for the prediction of the inhibitory activity of these peptides, the interaction with the molecular target at the active site is a positive result for considering a potential inhibitory effect. It is important to note that the docking experiments were performed with the SARS-CoV-1 3CLpro due to the nature of the co-crystallographic ligand (a pentapeptide without lateral substituents), whereas the SARS-CoV-2 3CLpro was used in the enzymatic and ligand fishing assays. However, the 3CLpros from both coronaviruses have an overall homology of 96% and the same amino acid residues at the active site (His-41 and Cys-145) and were therefore suitable for this analysis [8,27]. According to molecular docking data, all peptides were able to interact with both residues at the active site. Among them, PEP 4 stood out as the most promising one. Interactions via pi–pi stacking with His-41 and through hydrophobic interactions with Cys-145 are two of the main possible interactions regarding the 3CLpro active site and PEP 4. Additionally, PEP 4 was also able to interact via hydrogen bonds with multiple 3CLpro amino acid residues, such as Phe-140, Asn-142, Glu-166, Leu-167, Gln-189, Ala-191, and Gln-192. Generally, the higher the number of hydrogen bonds, the greater the stability of the complex is; in this case, the complex was 3CLpro-PEP 4 [28]. Furthermore, PEP 4 possibly interacts with fourteen of the fifteen amino acid residues implicated in the substrate binding. Taken together, these observations may explain the highest docking score of PEP 4 among the selected peptides (Figure 5C) [29].

It is worth noting that in this study, the full characterization of the fished-out ligands presents some limitations. Mass spectrometry (MS) is a very sensitive and robust analytical technique. However, collision-induced dissociation MS/MS analyses are often unable to differentiate isomers [30]. In this context, we could not distinguish the amino acids leucine and isoleucine in the peptide sequences. PEPs 4 and 7, for instance, presented the first (121.87) and the sixth (99.48) best scores among the screened peptides, respectively, and the difference between them is the amino acid isoleucine (PEP 4—EVIEFPLYIE) instead of leucine (PEP 7—EVLEFFKYLE) (Table 2). As a result, the molecular docking analysis may not have considered peptides actually present in the extract, thereby limiting our range of hypotheses. This underscores the importance of complementary approaches to MS, such as metastable atom-activated dissociation, which enables a more controlled fragmentation process and allows for differentiation between isomers like leucine and isoleucine. [31].

Finally, we evaluated the inhibitory effects of seven peptides identified through the ligand-fishing strategy, testing each one individually. Four peptides (PEPs 2, 3, 4, and 6) exhibited over 45% inhibition of 3CLpro activity, with PEP 2 demonstrating the highest activity. These results highlight the efficacy of the ligand-fishing method in efficiently purifying enzyme inhibitors from complex mixtures. Moreover, this technique reduces the use of organic solvents, promoting a more sustainable approach to drug discovery.

## 4. Materials and Methods

### 4.1. Seaweed Material

*Fucus ceranoides* L. 1753 (Ochrophytina, Fucaceae) samples were collected in January 2023 from the littoral zone of Arradon (GPS: 47°36′46.3″ N 2°50′08.7″ W) in Brittany (France).

### 4.2. Fucus ceranoides L. Extraction

The seaweed samples were washed with tap water, scraped, and drained to remove adherent seawater, sediment, and epiphytes. After cleaning, they were rinsed, wrung out, bench-dried at 20 °C, and ground into pieces measuring 3 mm with a hammermill. A hydroalcoholic extract of *F. ceranoides* (FCHE) was prepared by the ultrasonication (50 W, 30 kHz, UP50H Hielscher, Teltow, Germany) for 1 h at 20 °C of 1 g of dried material in 50 mL of ethanol 80% (Carlo Erba Reagents, Val-de-Reuil, France). The extract was filtered, transferred to a round-bottom flask, and dried using rotary evaporation (45 °C, 72 mbar, Büchi, Flawil, Switzerland). After the solvent evaporated, 198 mg of the FCHE was recovered and solubilized in 19.8 mL of DMSO (VWR International S.A.S, Briare, France) to obtain a 10 mg·mL^−1^ solution for further biological evaluation.

### 4.3. SARS-CoV-2 3CL_pro_ Enzymatic Assays

The enzymatic activity of the 3CLpro was measured following the hydrolysis of the peptidic chromogenic substrate TSAVLQ-pNA (both purchased from Sigma Aldrich, St. Louis, MO, USA), which releases pNA absorbing at 405 nm.

In a first step, the enzymatic activity of the commercial 3CLpro was checked via a kinetic hydrolysis study using a microplate assay to set the best substrate and enzyme concentrations. All assays were conducted at 30 °C in a 96-well plate (Nunc, Thermo Fischer Scientific, Bordeaux, France) with a final volume of 100 μL using HEPES as an assay buffer (25 mM, 0.2% *v*/*v* of Tween 20, and a pH of 7). The 3CLpro (200 μg) was resuspended with Milli-Q water (5.917 mL to give a 1 μM solution), divided into aliquots, and stored at −20 °C. Then, 50 μL of the 1 μM 3CLpro solution was first added to the wells and left to equilibrate for 5 min at 30 °C inside the microplate reader. Then, 50 μL of the substrate solution was added to the wells, starting the reaction. For the kinetic study, six different concentrations of the substrate (0, 62.5, 125, 250, 500, and 1000 μM) and one of the 3CLpro (500 nM) were studied in triplicate independent assays (n = 3). Absorbance was measured every minute for 10 min, and the initial enzymatic rate (V_0_) was determined using a microplate reader (SPECTROstar Nano, BMG Labtech, Champigny-sur-Marne, France). Finally, we standardized the concentration of the chromogenic substrate at 125 μM and set the 3CLpro concentration to 500 nM for all subsequent inhibition assays.

### 4.4. FCHE Inhibitory Effects against 3CLpro

For the inhibition assays, the 10 mg·mL^−1^ FCHE solution was half-diluted in the buffer to obtain a 5 mg·mL^−1^ solution containing 50% DMSO. Then, 2 µL of this solution was mixed with 48 µL of 1 μM 3CLpro and preincubated for 30 min with the enzyme before adding 50 µL of a 250 μM substrate solution. Thus, the final concentrations of the 3CLpro, FCHE, and substrate in the assay were 500 nM, 125 μM, and 100 µg·mL^−1^ (containing 1% DMSO), respectively. Additionally, 1 μM PF-00835231 (Sigma Aldrich, Saint-Quentin-Fallavier, France) was used as a reference inhibitor. Then, the initial velocities of each condition were normalized in relation to the control conditions (preincubation with 1% DMSO) to determine the percentage of enzymatic inhibition using Equation (1):

%Inhibition = (V_0 control_ − V_0 treatment_)/ V_0 control_ × 100, (1)

where V_0 control_ is the reference initial enzymatic rate and V_0 treatment_ is the enzymatic rate of the 3CLpro after incubation with the extract [32].

### 4.5. 3CL_pro_ Immobilization on N-Hydroxysuccinimide (NHS)-Functionalized Magnetic Particles

Immobilization of the 3CLpro on NHS-functionalized magnetic particles was performed according to the supplier recommendations (ThermoFisher Scientific, Waltham, WA, USA). Briefly, 300 μL of a suspension of 1 µm diameter magnetic particles (Pierce™ NHS-Activated Magnetic Beads, 10 mg·mL^−1^ in Dimethylacetamide) were mixed with 1 mL of ice-cold HCl 1 mM in a glass tube, vortexed and magnetically separated in a magnetic stand (DynaMag™-5 Magnet). The supernatant was discarded and the 3CLpro solution (200 μg in 300 μL of Milli-Q water) was immediately added and incubated under gentle agitation for 1 or 2 h for enzyme grafting. After these periods, the supernatants were collected following magnetic separation and stored at −20 °C for future analysis (of the unbound fraction of the 3CLpro). The resulting beads were washed two times with 1 mL of glycine solution (0.1 M, pH 2) and one time with 1 mL of Mill-Q water. Then, 1 mL of ethanolamine solution (3 M, pH 9) was added to the beads and incubated for 2 h under rotation. These steps were intended to saturate unoccupied enzyme binding sites with small amines with no enzymatic activity and avoid the interaction of remaining NHS functions with amines in the extract. Finally, the magnetic particles grafted with the 3CLpro (3CL-Pro-MPs) were recovered and rinsed twice with 1 mL of HEPES buffer before being stored at 4 °C in 1 mL of HEPES buffer containing 0.05% *w*/*v* of sodium azide. Before their use in the ligand-fishing experiments, the 3CLpro-MPs were rinsed two times with HEPES buffer to eliminate residual sodium azide. The final concentration of the 3CLpro-MP suspension was 3 mg·mL^−1^. In our study, we estimated the concentrations of grafted 3CLpro to be 63.5% and 67.7% of the initial mass (200 μg) after 1 and 2 h of incubation, resulting in 127 μg and 135.4 μg, respectively. We chose to proceed with the 3CLpro-MPs incubated for 2 h. Assuming that 135.4 μg of 3CLpro is immobilized on 3 mg of magnetic particles (corresponding to 300 μL of a 10 mg·mL^−1^ suspension), and given that the same enzyme concentration was required for both the 3CLpro-MPs and free 3CLpro (500 nM), we determined a need for 12.5 μL of the 3CLpro-MP suspension (equivalent to 1.69 μg of 3CLpro) in each well, with a final volume of 100 μL.

### 4.6. Determination of Immobilization Efficiency

The immobilization efficiency was determined using the difference in enzyme concentration (in moles) between the beginning and the end of the grafting procedure (the unbound fraction of the 3CLpro) after one or two hours of incubation. The number of moles of the 3CLpro in the unbound fraction was determined by correlating the optic density of the samples corrected by the dilution factor (0.1) and the concentration of the 3CLpro with the aid of a standard curve. Absorbance readings were performed in 1 mL quartz cuvettes at 279 nm. Equation (2) below was used to calculate the percentage of the 3CLpro immobilized on the surfaces of the beads:

%Immobilization = (n_1_ − n_2_)/ n_1_ × 100, (2)

where n_1_ is the initial number of moles of the 3CLpro and n_2_ is the number of moles in the unbound fraction of the 3CLpro [33].

### 4.7. Enzymatic Activity of 3CLpro-Grafted Magnetic Particles (3CLpro-MPs)

Verification of the enzymatic activity of the 3CLpro-MPs was carried out as previously described. The 3CLpro-MPs were added to the wells to a final concentration of 500 nM of the 3CLpro in a final volume of 100 μL (corresponding to 12.5 μL of 3CLpro-MPs). The enzymatic activity of the MPs alone was also checked as a negative control. After the equilibration period, the chromogenic substrate was added to a final concentration of 125 μM in the wells, initiating the enzymatic reaction. Then, the initial enzymatic rate (V_0_) of the 3CLpro-MPs was determined and normalized in relation to the V_0_ of the free 3CLpro.

### 4.8. Fishing the Chromogenic Substrate (TSAVLQ-pNA) as Proof of Concept of the Ligand-Fishing Procedure

The FCHE was voluntarily doped with 10 μM of the chromogenic substrate TSAVLQ-pNA in order to validate the fishing ability of the 3CLpro-MPs. The recovery rate was determined by HRMS after the fishing procedure.

### 4.9. Ligand Fishing of 3CLPro Inhibitors in FCHE Using 3CLpro-MPs

**Figure 7:**
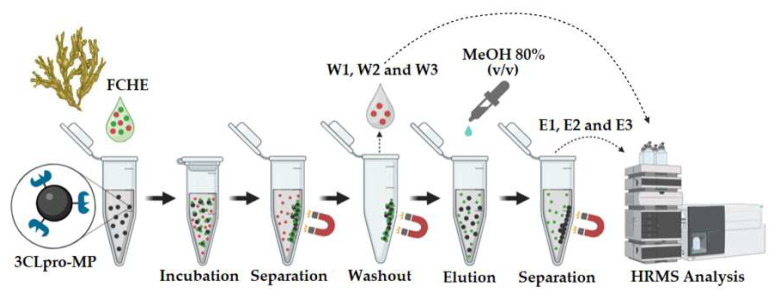
FCHE ligand-fishing procedure. Adapted from [35].

For the ligand-fishing assay, 50 μL of 3CLpro-MPs were mixed with 20 μL of the FCHE and 130 µL of a buffer. The FCHE final concentration was 1 mg·mL^−1^, and the mix was incubated for 2 h. The DMSO concentration (10%, *v*/*v*) was checked to ensure its compatibility with the enzymatic activity [34]. After incubation for 2 h at 20 °C, the beads were recovered with the aid of a magnetic stand, rinsed three times with HEPES buffer to wash out weakly bound ligands (W1, W2, and W3), rinsed two times with Milli-Q water to rinse away residual buffer, and rinsed three times with methanol 80% *v*/*v* to elute strongly bound ligands (E1, E2, and E3). In the end, W1, W2, W3, E1, E2, and E3 were stored at −20 °C for analysis using ultra-high performance liquid chromatography coupled to high-resolution mass spectrometry and ultraviolet spectrometry (UPLC-MS and UPLC-UV). A simplified scheme is provided in Figure 7.

### 4.10. Ultra-Performance Liquid Chromatography Coupled to Ultraviolet Spectrometry and High-Resolution Mass Spectrometry (UPLC-DAD-MS/MS)

For this analysis, an Acquity UPLC H-class system (Waters, Milford, CT, USA) coupled to a photodiode array (Waters 2996) and a high-resolution mass spectrometer (an XEVO G2S Q-TOF equipped with an electrospray ionization source (Waters, Manchester, UK)) was used. For injection, all samples were dried, resuspended in 200 μL of 70% (*v*/*v*) solvent A (H_2_O + 0.1% *v*/*v* formic acid) and 30% (*v*/*v*) solvent B (acetonitrile 0.1% *v*/*v* formic acid), centrifuged at 13,000 RPM for 5 min, and filtered using a PVDF filter (0.22 μm). Then, 5 μL of each sample was injected into the equipment for MS, MS/MS, and UV analyses. For UPLC, a BEH C_18_ column (2.1 × 150 mm; 1.7 μm) was used, and the temperature of the analysis was kept at 40 °C. The gradient for solvents A and B was as follows: 0–3 min, 70% A and 30% B; 3–4 min 20% A and 80% B; 4–6 min 20% A and 80% B; 6–6.5 min, 0% A and 100% B; 6.5–10 min 0% A and 100% B; 10–10.1 min, 70% A and 30% B; and 10.1–15 min, 70% A and 30% B. Mass spectrometry data were acquired in both polarities (capillary voltages of +3 kV and −2.5 kV) using an ESI-QToF setup, with *m*/*z* ranging from 50 to 1200 and scan time of 0.1 s. An MSMS analysis was conducted based on an ion intensity threshold of 200,000.s^−1^, and a UV analysis was performed in a detection range from 250 to 800 nm and 10 spectra.s^−1^.

### 4.11. Peptide Sequence Determination

The peptides’ sequences were manually determined using a de novo approach. After establishing a sequence through a series of b or y ions, complementary series were annotated and verified using the freely accessible proteomics toolkit (http://db.systemsbiology.net/proteomicsToolkit/FragIonServlet.html (accessed on 16 January 2024)). Finally, all peptide sequences were assigned according to the nomenclature established by Roepstorff and Fohlman [36].

### 4.12. 3CLpro Inhibition by the Mix of Eluates (E1 + E2 + E3) Obtained through the Ligand-Fishing Assay

After the HRMS analysis, the three eluates were concentrated at room temperature and resolubilized as a mix in 100 μL of HEPES buffer for the enzymatic assay, which was conducted as previously described. Briefly, 500 nM of the 3CLpro was incubated for 30 min with 20 μL of the mix of eluates (E1 + E2 + E3). Then, 125 μM of the chromogenic substrate was added to the well, initiating the enzymatic reaction.

### 4.13. Peptide Synthesis and Inhibitory Activity

Peptides identified through the ligand-fishing purification process were synthesized by the company SB-PEPTIDE (Saint Egreve, France, https://www.sb-peptide.com/ (accessed on 24 April 2024) as TFA salts with a purity index superior to 95% (1 mg). The peptides’ purity was checked by HRMS (data not shown). The inhibitory activity of each peptide was tested at 10 µM following the 3CL-Pro inhibition assay described in Section 4.4.

### 4.14. Dynamic Light Scattering (DLS) and Zeta Potential Analysis

Low-volume polystyrene cuvettes were used for the DLS assay. The zeta potential was determined using folded capillary cells. A Zeta Sizer Ultra equipment (Malvern Instrument, France) was used to conduct both analyses. All measures were performed in triplicate with 10 s of equilibration at 25 °C.

### 4.15. Molecular Docking

Molecular docking calculations were performed using the GOLD 2022.3.0 package [37]. The score function used was ChemPLP due to its superior performance compared to the other functions available in the package, both in pose prediction and virtual screening [38]. The calculations were performed by centering the box on the co-crystallographic ligand, with X = −24.352, Y = −37.061, and Z = 3.926 (Figure 7). The crystallographic structure chosen for the calculations was the PDB code 3AW0 (Resolution = 2.30 Å), downloaded from the Protein Data Bank (www.rcsb.org (accessed on 22 December 2023), which corresponds to the SARS-CoV 3CLpro complexed with a co-crystallized peptide aldehyde ligand is structurally close to peptides fished in the present study [26]. The removal of the co-crystallographic ligands and the addition of hydrogens were performed using Chimera software [39]. The 3D structures of the peptides were obtained based on amino acid sequencing using PEP-FOLD 2.0, a feature of the RPBS (Ressource Parisienne en Bioinformatique Structurale) portal [40]. The adjustment of the protonation state to a pH of 7 was performed in Chimera for the peptides and using the online platform APBS (Adaptive Poisson-Boltzmann Solve) for the 3CLpro [41]. The peptides assessed through this methodology were chosen according to three main criteria: the peptides should have at least six amino acids; their sequences should have been fully determined by HRMS (except for Ile/Leu since they have the same mass), and they should present a majority of hydrophobic amino acids due to their importance for anti-3CLpro activity [42].

### 4.16. Statistical Analysis

Experiments were conducted independently in triplicate. Parametric data are expressed as mean ± SD values. Data normalization was performed in relation to the enzymatic activity of the free 3CLpro (100% enzymatic activity) and is expressed as the mean ± SD. Comparisons between two groups was performed using Student’s *t*-test. A one-way ANOVA followed by Dunnett’s multiple-comparison test was performed for the inhibition assay with the isolated peptides (PEPs 1-7) and the control condition. The difference between groups was considered statistically significant when *p* < 0.01.

## 5. Conclusions

This study is the first to document *Fucus ceranoides* peptides exhibiting potent inhibitory effects on the SARS-CoV-2 3CL protease by binding with a high affinity for its proteolytic active site. These findings lay the groundwork for potential future developments in antiviral preventive medical formulations incorporating a hydroalcoholic extract of *Fucus ceranoides* or its isolated protease-inhibiting peptides. Finally, this work confirms the relevance of the ligand-fishing strategy for the fast and efficient purification of enzyme ligands and inhibitors from complex seaweed matrices.

## Figures and Tables

**Table 1:** Values of PDI, zeta potential and mean particle size for MP and 3CLpro-MP. Data are expressed as mean ± SD values.

	PDI	Zeta Potential (mV)	Mean Particle Size (nm)
**MP**	0.1622 ± 0.026	−35.06 ± 0.493	1311.72 ± 6.58
**3CLpro-MP**	0.1298 ± 0.058	−35.06 ± 0.321	1535.65 ± 5.12

**Table 2:** Docking scores for the co-crystallographic peptide ligand and 3CLpro-MP-fished peptides.

Ligand	Score
Co-crystallographic peptide ligand (Ac-SAVLH)	83.11
PEP 1 (VVGVVVY)	93.91
PEP 2 (VEIEFFKY)	113.37
PEP 3 (VELEFFKY)	100.73
PEP 4 (EVIEFFKYIE)	121.87
PEP 5 (EVLEFFKYIE)	106.07
PEP 6 (EVIEFFKYLE)	103.18
PEP 7 (EVLEFFKYLE)	99.48

## Data Availability

Data will be made available upon request.

## Supplementary Materials

The following supporting information can be downloaded at https://www.mdpi.com/article/10.3390/md22060244/s1, Figure S1: MS/MS fragmentation profile of PEP 1 (Val-Val-Gly-Val-Val-Val-Tyr) with fragment ions from b series in blue and y series in red; Figure S2: MS/MS fragmentation profile of PEP 2/PEP 3 (Val-Glu-Ile-Glu-Phe-Phe-Lys-Tyr/Val-Glu-Leu-Glu-Phe-Phe-Lys-Tyr) with fragment ions from b series in blue, y series in red, and a series in green; Figure S3: Figure S3—MS/MS fragmentation profile of PEP 4-7 (Glu-Val-Ile-Glu-Phe-Phe-Lys-Tyr-Ile-Glu/Glu-Val-Leu-Glu-Phe-Phe-Lys-Tyr-Ile-Glu/Glu-Val-Ile-Glu-Phe-Phe-Lys-Tyr-Leu-Glu/Glu-Val-Leu-Glu-Phe-Phe-Lys-Tyr-Leu-Glu) with fragment ions from b series in blue, y series in red, and a series in green; Figure S4: Most stable conformation of PEP 1 at (A) Van der Waals surface, (B) binding site of co-crystallized peptide aldehyde ligand with highest docking score, and (C) 2D representation of possible interactions.; Figure S5: Most stable conformation of PEP 2 at (A) Van der Waals surface, (B) binding site of co-crystallized peptide aldehyde ligand with highest docking score, and (C) 2D representation of possible interactions; Figure S6: Most stable conformation of PEP 3 at (A) Van der Waals surface, (B) binding site of co-crystallized peptide aldehyde ligand with highest docking score, and (C) 2D representation of possible interactions; Figure S7: Most stable conformation of PEP 5 at (A) Van der Waals surface, (B) binding site of co-crystallized peptide aldehyde ligand with highest docking score, and (C) 2D representation of possible interactions; Figure S8: Most stable conformation of PEP 6 at (A) Van der Waals surface, (B) binding site of co-crystallized peptide aldehyde ligand with highest docking score, and (C) 2D representation of possible interactions. Figure S9: Most stable conformation of PEP 7 at (A) Van der Waals surface, (B) binding site of co-crystallized peptide aldehyde ligand with highest docking score, and (C) 2D representation of possible interactions; Figure S10: Hydrodynamic size distribution of magnetic particle alone (MP) and magnetic particle with immobilized 3CLpro (3CLpro-MP).
