# Supplementary material for: The Identification of Peptide Inhibitors of the Coronavirus 3CL Protease from a Fucus ceranoides L. Hydroalcoholic Extract Using a Ligand-Fishing Strategy
Source: Mar Drugs. 2024 May 27;22(6):244. doi: 10.3390/md22060244 (PMC11205194; doi:10.3390/md22060244)
Supplement: Supplementary file 1 [file marinedrugs-22-00244-s001.zip › marinedrugs-2852279-supplementary.pdf]

Supplementary material - Identification of peptide inhibitors of the coronavirus 3CL protease from *Fucus ceranoides* L. hydroalcoholic extract using a Ligand-fishing strategy

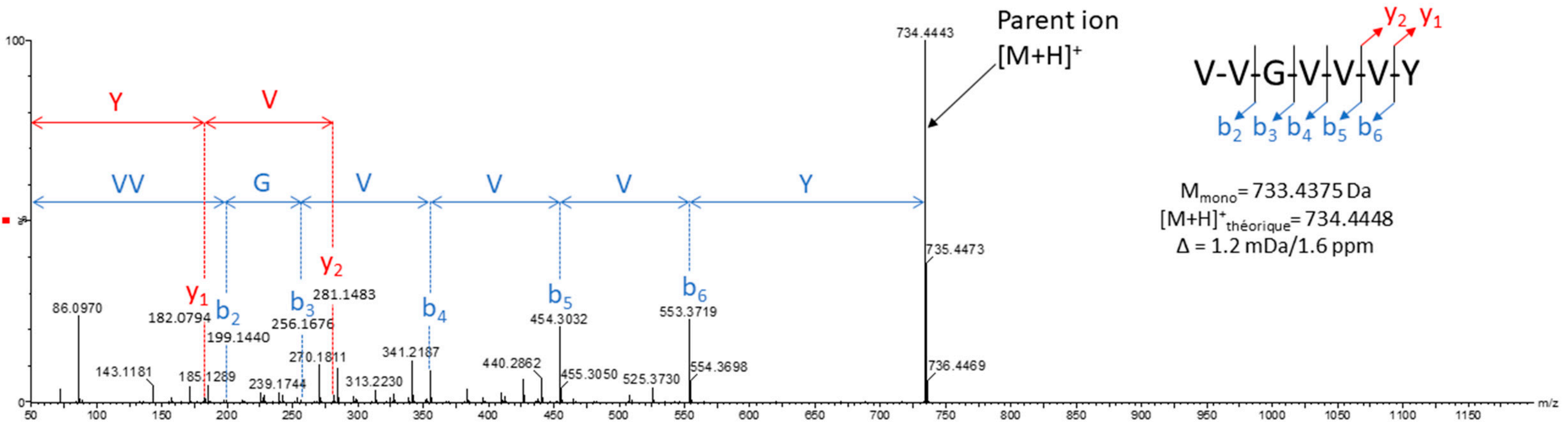

Figure S1 – MS/MS fragmentation profile of PEP 1 (Val-Val-Gly-Val-Val-Val-Tyr) with fragment ions from b series in blue and y series in red.

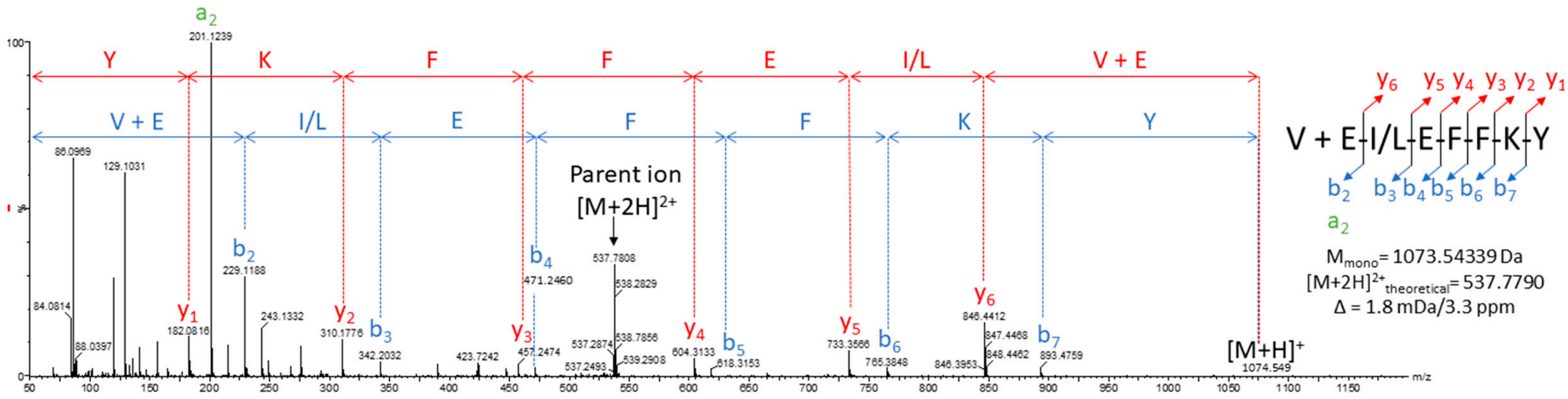

Figure S2 – MS/MS fragmentation profile of PEP 2/PEP 3 (Val-Glu-Ile-Glu-Phe-Phe-Lys-Tyr/Val-Glu-Leu-Glu-Phe-Phe-Lys-Tyr) with fragment ions from b series in blue, y series in red, and a series in green.

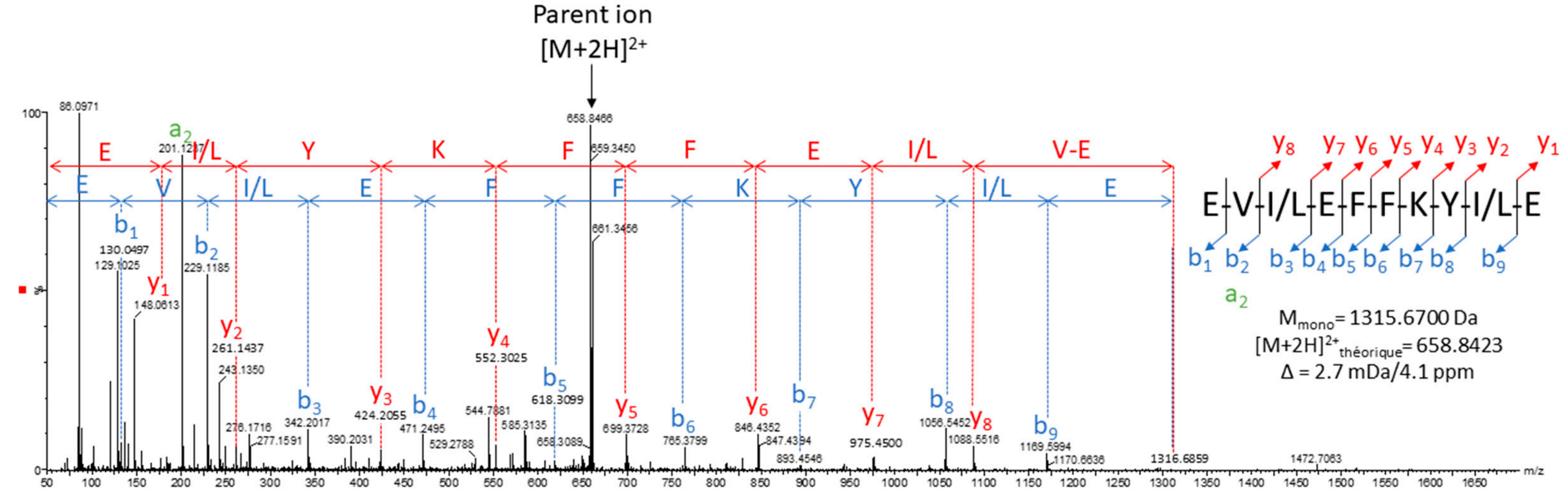

Figure S3 – MS/MS fragmentation profile of PEP 4-7 (Glu-Val-Ile-Glu-Phe-Phe-Lys-Tyr-Ile-Glu/Glu-Val-Leu-Glu-Phe-Phe-Lys-Tyr-Ile-Glu/Glu-Val-Ile-Glu-Phe-Phe-Lys-Tyr-Leu-Glu/Glu-Val-Leu-Glu-Phe-Phe-Lys-Tyr-Leu-Glu) with fragment ions from b series in blue, y series in red, and a series in green.

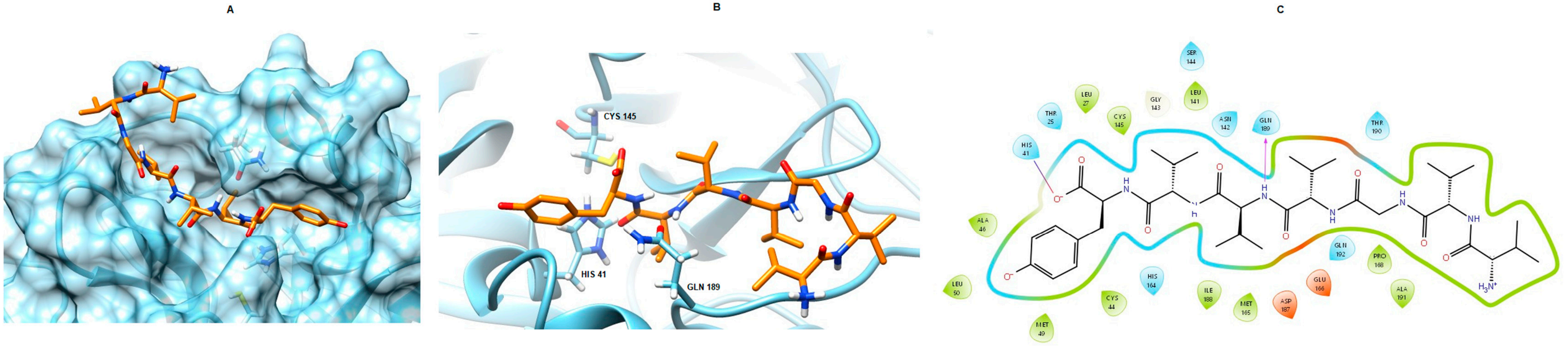

Figure S4 – Most stable conformation of PEP 1 in (A) Van der Waals surface, (B) binding site of 3AW0, and (C) 2D representation of possible interactions.

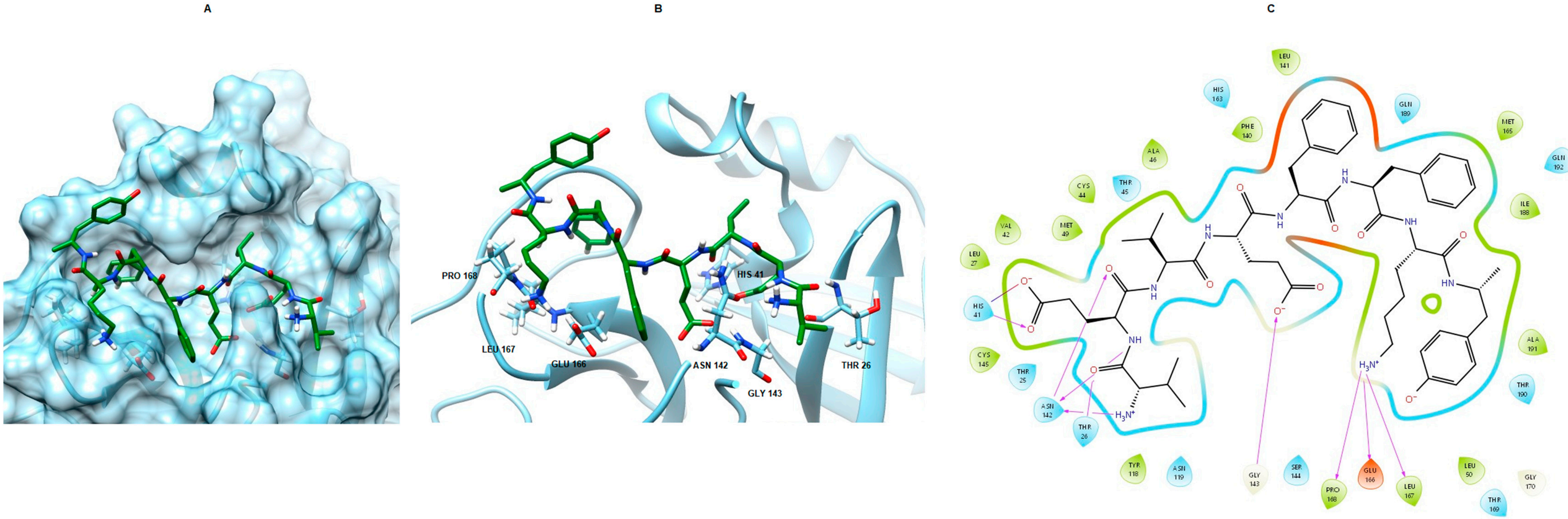

Figure S5 – Most stable conformation of PEP 2 in (A) Van der Waals surface, (B) binding site of 3AW0, and (C) 2D representation of possible interactions.

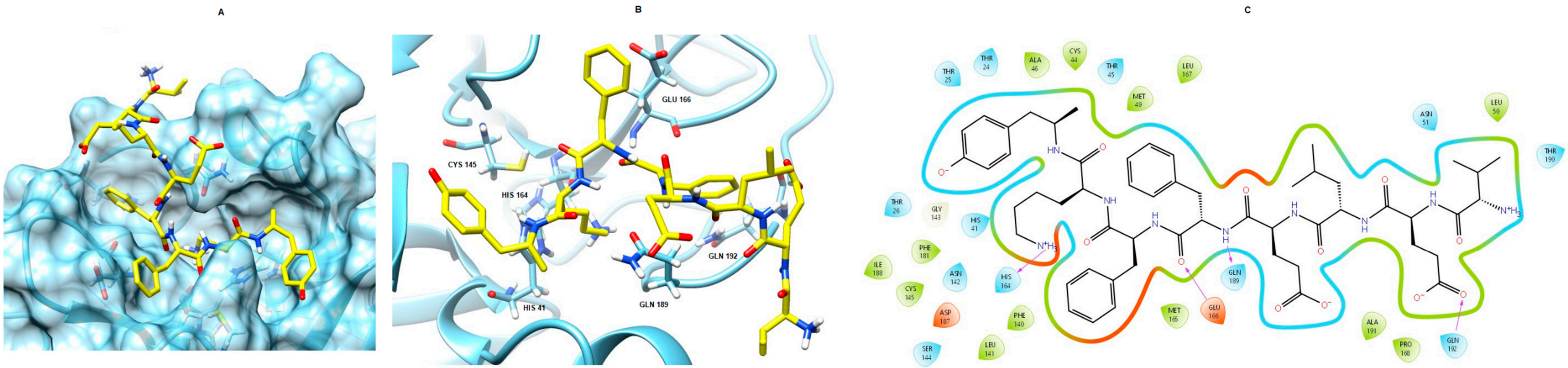

Figure S6 – Most stable conformation of PEP 3 in (A) Van der Waals surface, (B) binding site of 3AW0, and (C) 2D representation of possible interactions.

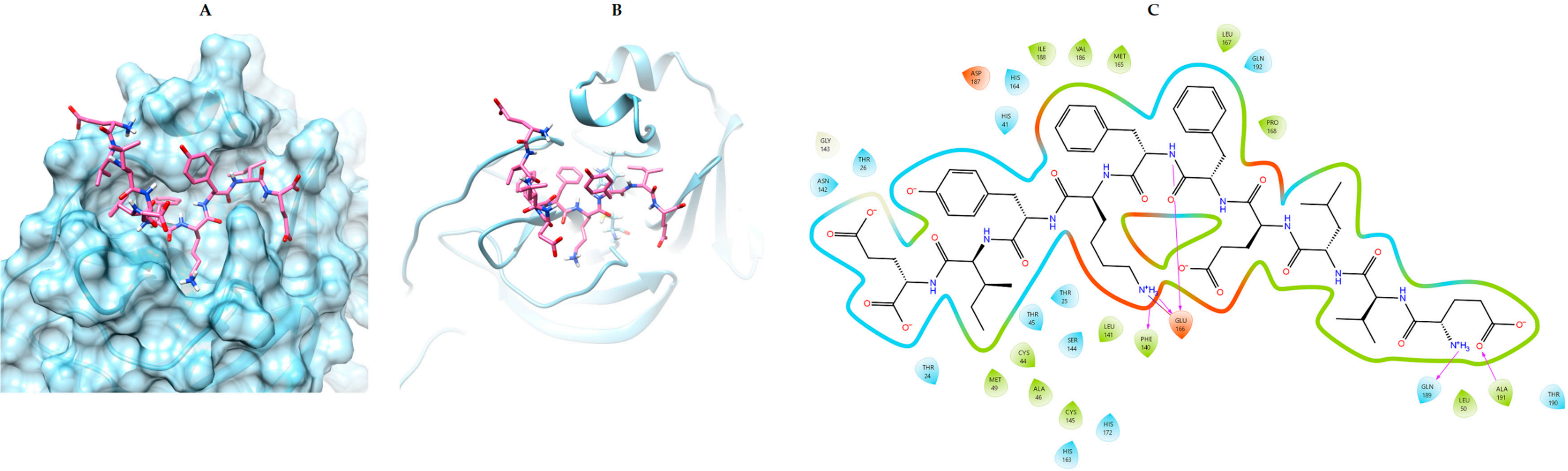

Figure S7 – Most stable conformation of PEP 5 in (A) Van der Waals surface, (B) binding site of 3AW0, and (C) 2D representation of possible interactions.

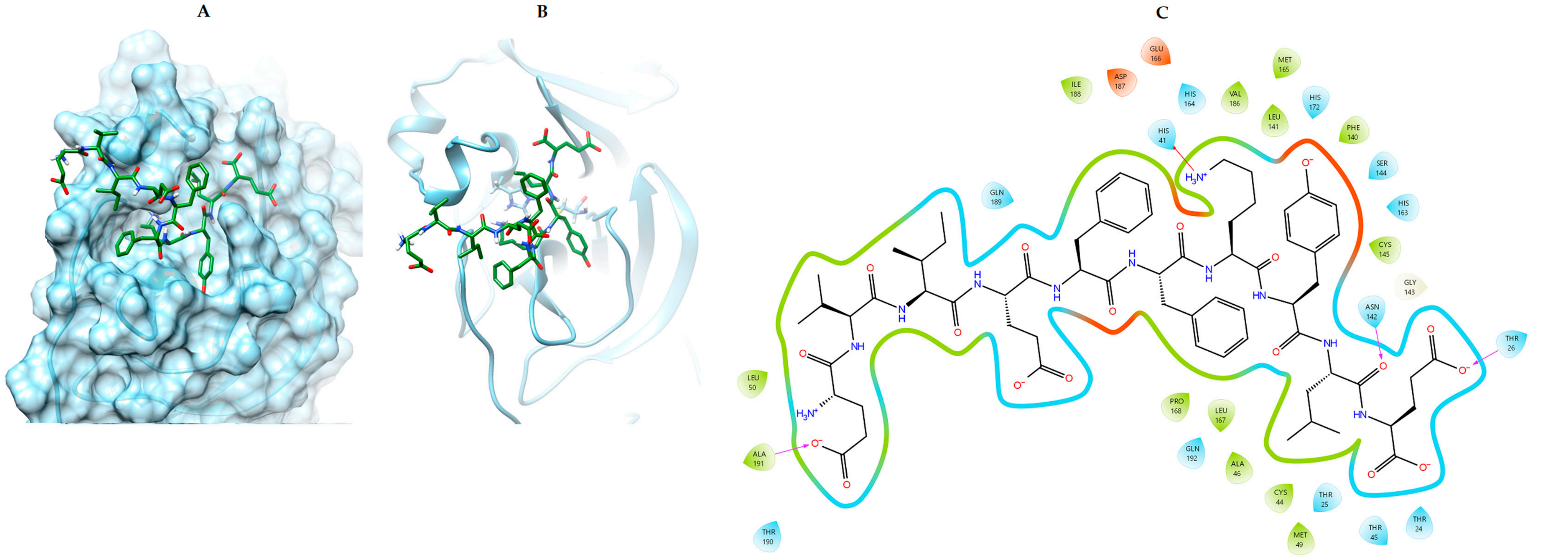

Figure S8 – Most stable conformation of PEP 6 in (A) Van der Waals surface, (B) binding site of 3AW0, and (C) 2D representation of possible interactions.

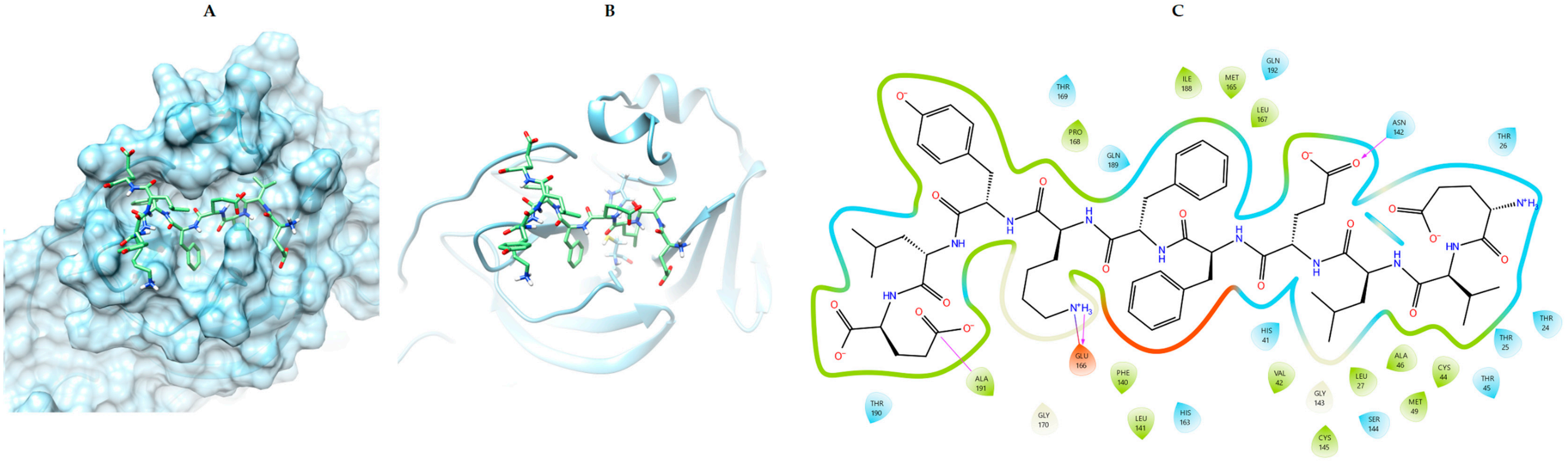

Figure S9 – Most stable conformation of PEP 7 in (A) Van der Waals surface, (B) binding site of 3AW0, and (C) 2D representation of possible interactions.

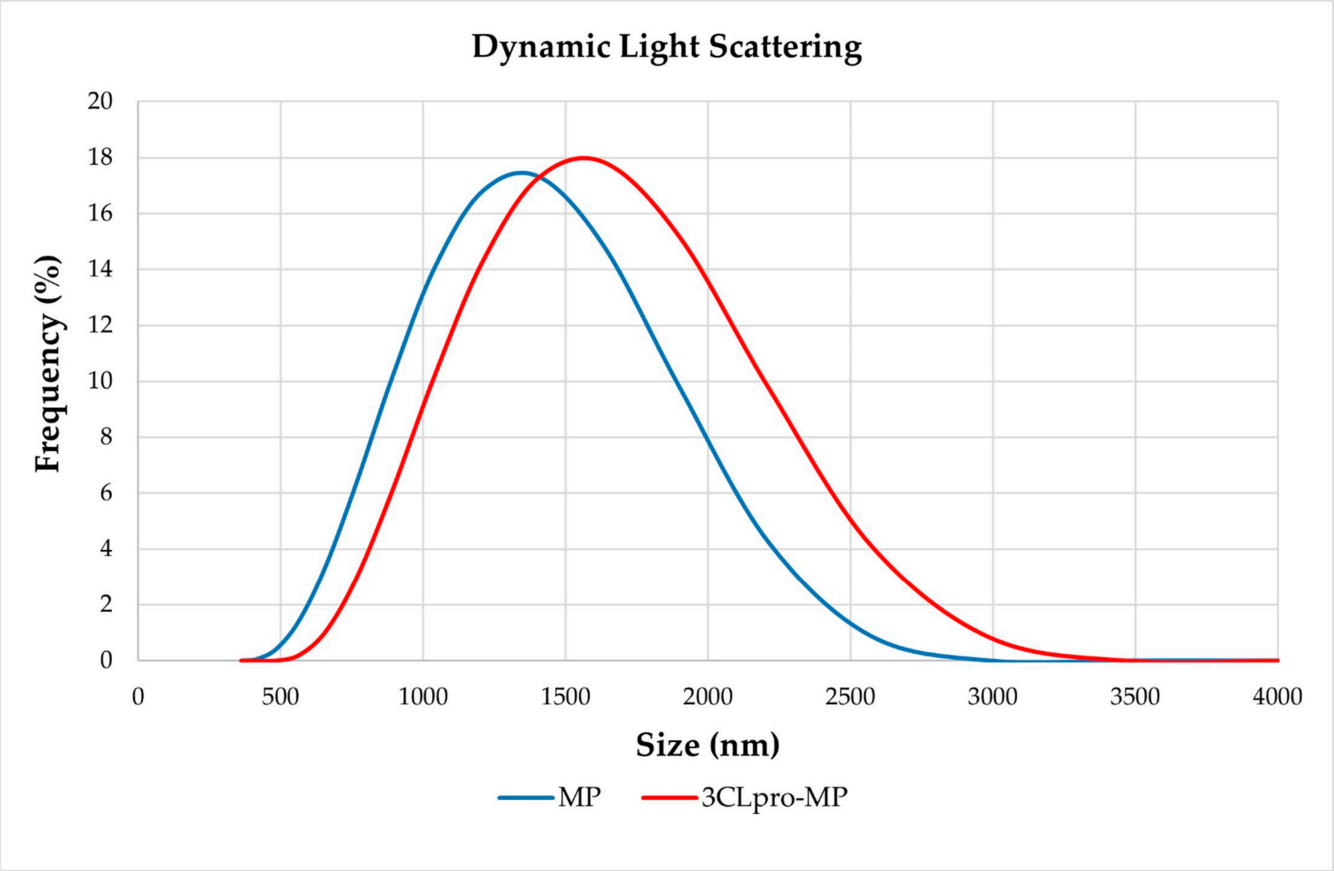

Figure S10 – Hydrodynamic size distribution of the magnetic particle alone (MP) and the magnetic particle with immobilized 3CLpro (3CLpro-MP).
